# Formulation and Optimization of Avanafil Biodegradable Polymeric Nanoparticles: A Single-Dose Clinical Pharmacokinetic Evaluation

**DOI:** 10.3390/pharmaceutics12060596

**Published:** 2020-06-26

**Authors:** Hibah M. Aldawsari, Usama A. Fahmy, Fathy Abd-Allah, Osama A. A. Ahmed

**Affiliations:** 1 Department of Pharmaceutics, Faculty of Pharmacy, King Abdulaziz University, Jeddah 21589, Saudi Arabia; uahmedkauedu.sa@kau.edu.sa (U.A.F.); oaahmed@kau.edu.sa (O.A.A.A.); 2 Department of Pharmaceutics and Industrial Pharmacy, Faculty of Pharmacy, Al-Azhar University, Cairo 11865, Egypt; fathyfet@yahoo.com; 3 Department of Pharmaceutics & Industrial Pharmacy, Faculty of Pharmacy, Minia University, Minia 61111, Egypt

**Keywords:** biodegradable polymers, Box–Behnken, drug delivery, nanoparticles, clinical pharmacokinetics

## Abstract

Avanafil (AVA) is a second-generation phosphodiesterase-5 (PDE5) inhibitor. AVA shows high selectivity to penile tissues and fast absorption, but has a bioavailability of about 36%. The aim was to formulate and optimize AVA-biodegradable nanoparticles (NPs) to enhance AVA bioavailability. To assess the impact of variables, the Box–Behnken design was utilized to investigate and optimize the formulation process variables: the AVA:poly (lactic-co-glycolic acid) (PLGA) ratio (*w*/*w*, X1); sonication time (min, X2); and polyvinyl alcohol (PVA) concentration (%, X3). Particle size (nm, Y1) and EE% (%, Y2) were the responses. The optimized NPs were characterized for surface morphology and permeation. Furthermore, a single-oral dose (50 mg AVA) pharmacokinetic investigation on healthy volunteers was carried out. Statistical analysis revealed that all the investigated factors exhibited a significant effect on the particle size. Furthermore, the entrapment efficiency (Y2) was significantly affected by both the AVA:PLGA ratio (X1) and PVA concentration (X3). Pharmacokinetic data showed a significant increase in the area under the curve (1.68 folds) and plasma maximum concentration (1.3-fold) for the AVA NPs when compared with raw AVA. The optimization and formulation of AVA as biodegradable NPs prepared using solvent evaporation (SE) proves a successful way to enhance AVA bioavailability.

## 1. Introduction

**Figure 1:**
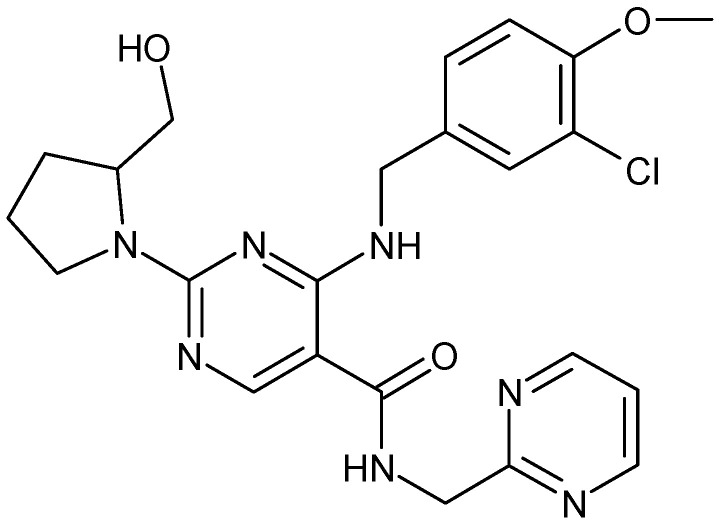
Chemical structure of AVA.

Avanafil (AVA, Figure 1) is a drug recently approved in the United States and Europe [1,2]. AVA is a second-generation phosphodiesterase type 5 inhibitor (PDI5) used for erectile dysfunction [3,4]. AVA is characterized by its faster onset and improved selectivity compared with other PDI-class drugs. AVA showed a more favorable profile against other enzymes known to be affected by this class of drugs, especially PDE6, where AVA was more than 100 times less potent than the PDE5. Therefore, AVA had a low propensity for visual disturbances [1,2,5,6,7]. AVA displayed higher selectivity for PDE5 versus PDE6 (121-fold) compared to sildenafil (16-fold). AVA shows time for maximum plasma concentration (T_max_) at around 30–45 min. The mean half-life (t_1/2_) ranges from very short half-lives to up to 17 h, as a result of the influence of food intake in delaying AVA absorption. AVA solubility is pH-dependent, as it is practically insoluble in neutral and alkaline pH, while it is soluble in an acidic medium. AVA is a Class I or Class II Biopharmaceutics Classification System (BCS), according to the dose administered compared to its solubility value at pH 7 [8]. Accordingly, trials to improve AVA bioavailability should focus on the improvement of AVA water solubility and permeation characteristics. One of the approaches applied is achieved by particle size reduction to the nanoscale range through the nanoparticulate formulation technique.

Poly (d,l-lactide-co-glycolide) (PLGA) has been approved by the Food and Drug Administration (FDA), and is highly suited as a biocompatible polymer for controlled drug release, as it is hydrolyzed to monomers of glycolic and lactic acid, both of which are easily degraded in the body. Researchers have used several techniques to encapsulate active substances in the PLGA matrix, such as spray drying, cryopreparation and emulsion-solvent evaporation techniques [9,10,11,12,13]. The first important parameter in this process was the selection of a suitable molecular weight (MW) from PLGA. AVA encapsulation using low MW polymers has been shown to result in a poor encapsulation performance [14]. Consequently, when we started designing a formula for AVA loaded with PLGA, we selected a MW of 30,000–60,000, due to its low cost compared to other high-MW PLGAs. Another important factor was the solubility of PLGA in widely used organic solvents such as chloroform and dichloromethane, ethyl acetate, acetone and others. PLGA’s solubility depends on the percentage of lactide and glycolide proportions. The increased proportion of lactide relative glycolide in a PLGA backbone leads to a reduction in the rate and onset of PLGA degradation [15]. On the other hand, increased glycolide proportion relative to the lactide one improves the PLGA hydration, and consequently enhances PLGA hydrolysis and degradation. The selected PLGA in this investigation was a lactide:glycolide ratio of 50:50 for modest degradation, to avoid the technical difficulties of the polymer’s solubility [11,16].

The emulsion solvent evaporation method, with subsequent modifications, is the most commonly utilized technique to encapsulate pharmaceutical substances [17,18,19]. AVA is soluble in dichloromethane (DCM), which makes the drug suitable for polymeric encapsulation using the single emulsion (SE) oil in water (o/w) technique [20,21]. This work aimed to develop and optimize AVA nanoparticle (NPs) formulation, with improved bioavailability in human volunteers. The SE method was applied for the preparation of AVA-NPs. The optimization of the prepared NPs achieved the smallest particle size with the maximum entrapment efficiency (EE%). Furthermore, investigating the clinical pharmacokinetic (PK) parameters of the optimized AVA-NPs formula compared with raw (pure) AVA was carried out on human volunteers.

## 2. Materials and Methods

### 2.1. Materials

AVA was procured from Jinlan Pharm-Drugs Technology Co., Ltd. (Hangzhou, China), while PLGA lactide:glycolide (50:50), mol wt 30,000–60,000, DCM, polyvinyl alcohol (PVA) Mw 89,000–98,000 and all other chemicals were from Sigma-Aldrich (St. Louis, MO, USA). The empty hard gelatin capsules utilized in the clinical investigation were donated by Capsugel (Lonza Company, Basel, Switzerland). 

### 2.2. Formulation of AVA Biodegradable Polymeric NPs

AVA-NPs were prepared using the SE technique with an AVA to PLGA weight ratio according to the proposed experimental design (Table 1). PLGA and AVA were dissolved in 2 mL DCM in screw-capped test tubes, and was then sonicated in an ice bath for 15 s using a probe sonicator (Hielscher, Ultrasound UP-50 H, Teltow, Germany) for a duration of 2–6 min at 40% amplitude and on-off pulse (20 s:10 s). The organic phase was added to 10 mL of PVA aqueous solution (according to the proposed experimental design), and then sonicated according to the proposed experimental design using a probe sonicator in an ice bath to obtain the o/w emulsion. The formed emulsion was then added to 20 mL of 0.25% PVA solution, and stirred for 4 h until the evaporation of DCM. The formed NPs were separated by centrifugation at 20,000 rpm for 40 min using a Sigma 3-30KS refrigerated centrifuge (Sigma Laboratory Centrifuges, Osterode am Harz, Germany), washed twice with distilled water and centrifuged, and then freeze-dried for 24 h. 

### 2.3. Investigating the Effect of Formulation and Process Parameters Using the Box–Behnken Design

The Box–Behnken design was utilized to optimize the variables: the AVA:PLGA ratio (*w*/*w*, X1); sonication time (min, X2); and PVA concentration (%, X3). Both particle size (nm, Y1) and EE% (%, Y2) were selected as the responses, as shown in Table 1. Each variable was studied at three levels, and the experimental runs were prepared with all possible combinations, yielding a total of 17 formulations, as seen in Table 2. The observed responses were subjected to statistical analysis by an ANOVA test at a 95% level of significance using the Design-Expert^®^ Software Version 12 (Stat-Ease Inc., Minneapolis, MN, USA) to independently identify the significance of variables’ effects, and the possible interaction between them.

### 2.4. AVA-NPs Characterization

#### 2.4.1. Particle Size Analysis and Zeta Potential

AVA-NPs samples (10 mg) were diluted with 5 mL deionized water and vortexed for 1 min, and examined for particle size analysis and zeta potential using a Zetatrac^®^ analyzer (Microtrac Inc., Montgomeryville, PA, USA).

#### 2.4.2. AVA-NPs EE%

AVA-NPs EE% was investigated as previously reported [22]. Briefly, AVA in the formed PLGA NPs was analyzed by high-performance liquid chromatography (HPLC) equipped with a diode array detector (DAD) and set at 230 nm, with separation carried out by using a Thermo RP-C18 (250 mm × 4.6 mm, 4.5 µm particle size) column, a 20 μL injection volume, a mobile phase composed of 0.1 M Ammonium Acetate buffer, PH 2.5, and methanol and acetonitrile with ratios (20:40:40) [23], after dissolving the prepared AVA-NPs in methanol. AVA EE% was calculated by Equation (1):

(1) $$AVA\;EE\%=\left(\frac{AVA\;weight\;in\;the\;NPs}{AVA\;weight\;initially\;added}\right)\times 100$$

### 2.5. Optimization of AVA PLGA Nanoparticles

AVA PLGA NPs were optimized utilizing a numerical approach. The desirability function that amalgamates all the responses was computed to anticipate the optimized levels of the investigated independent variables. The criteria for the optimum formulation were set at minimizing the particle size and maximizing the EE%. The optimum formulation was then selected for subjection to further investigations.

### 2.6. Examination of Optimized AVA-NPs Morphology

The surface morphology of the prepared optimized AVA-NPs was investigated using scanning electron microscopy (SEM), after subjecting the prepared formulation to the lyophilization process. Optimized AVA-NPs were mounted on metal stubs, spattered with gold, and investigated by Jeol JSM 7600f filed emission SEM.

### 2.7. Optimized AVA-NPs In-Vitro Permeation Study

AVA permeation from the optimized NPs was studied using the Franz automated vertical-cell diffusion system (Hanson Research, MicroettePlus, Chatsworth, USA). The diffusion membrane was a 0.1 μm pore size nylon membrane (PALL Corporation, Port Washington, MI, USA). The prepared NPs were loaded between the diffusion system compartments. Samples were withdrawn at 0, 0.5, 1, 1.5 and 2 h, and the medium was 0.1 N HCl. After 2 h, the medium was changed to phosphate buffer saline (pH 6.8) stirred at 400 rpm, and samples were at 2.5, 3, 4, 6, 8, 10 and 12 h, and analyzed by HPLC. AVA sample concentrations were analyzed utilizing the same HPLC method used before in Section 2.4.2.

### 2.8. Single Dose Clinical Pharmacokinetic Investigation of AVA-PLGA NPs in Healthy Human Volunteers

The optimized AVA-NPs and raw AVA, filled in hard gelatin capsules, were investigated for their PK parameters. A single 50 mg AVA dose was administered orally with 250 mL of water by healthy male subjects (25 to 45 years of age). The design and method followed were previously reported [24]. The design was briefly open label, with one period that was carried out at the Egyptian Research and Development Company (ERDC), Cairo, Egypt. The study protocol was ethically approved by the ERDC Research Ethical Committee on 30 August 2017, to ensure agreement with the Declaration of Helsinki and the International Conference on “Harmonisation of Good Clinical Practices”.

The twelve male participants, eligible for the study through full medical examination and willing to participate in this clinical trial, provided written informed consent. They complied with the study requirements and were classified into two groups (six each): group I was orally administered the selected AVA-NPs, and group II was orally administered raw AVA. Both dosage forms were filled in soft gelatin capsules and swallowed with 250 mL of water. Blood samples (5 mL) were collected at 0, 0.08, 0.17, 0.25, 0.5, 0.75, 1, 1.25, 1.5, 2, 4, 6, 8, 10, 12 and 24 h, and were then centrifuged at 3500 rpm for 10 min (Centurion, West Sussex, UK) and stored at −80 °C.

### 2.9. AVA Human Plasma Analysis

The HPLC with MS/MS detection (HPLC-MS/MS) method was developed at ERDC laboratories for AVA analysis in human plasma. The method was validated according to the FDA Bio-analytical Method Validation Guidelines 2003. The method assay linearity for AVA was within the concentration range of 1–1000 ng/mL, with a regression coefficient (R^2^) = 0.997. The results were within the acceptance criteria as indicated in the recommended guidelines. The mean AVA recovery was 101 at 10 ng/mL (LLOQ) & 104.2% at 1000 ng/mL (ULOQ). The described method proved to be sensitive, accurate and reproducible, with a lower limit of AVA quantification of 1 ng/mL.

The HPLC-MS/MS-system consists of Agilent series 1200, Agilent Technologies, with a quaternary pump (G1311A), autosampler (G1329A) and vacuum degasser (G1322A). The mobile phase was acetonitrile 50% and ammonium formate 10 mmole 50%, at a flow rate of 6 mL/min, and the reverse phase column Intersil ODS-3 (4.6 mm × 50 cm, dp 5 µm Sigma–Aldrich) at 25 °C. Retention time was 2.3 min for AVA and 2.7 for vardenafil (internal standard).

### 2.10. Pharmacokinetic Data Analysis

A non-compartmental model was utilized for AVA plasma data analysis. Maximum AVA plasma concentration (C_max_), T_max_, the area under the plasma AVA concentration-time curve (AUC), the elimination rate constant (k_e_), half-life (t_1/2_) and mean residence time (MRT) were performed, and the significance of data difference was carried out using unpaired *t*-test (two-tailed). The confidence level was set at *p* < 0.05.

## 3. Results

**Figure 2:**
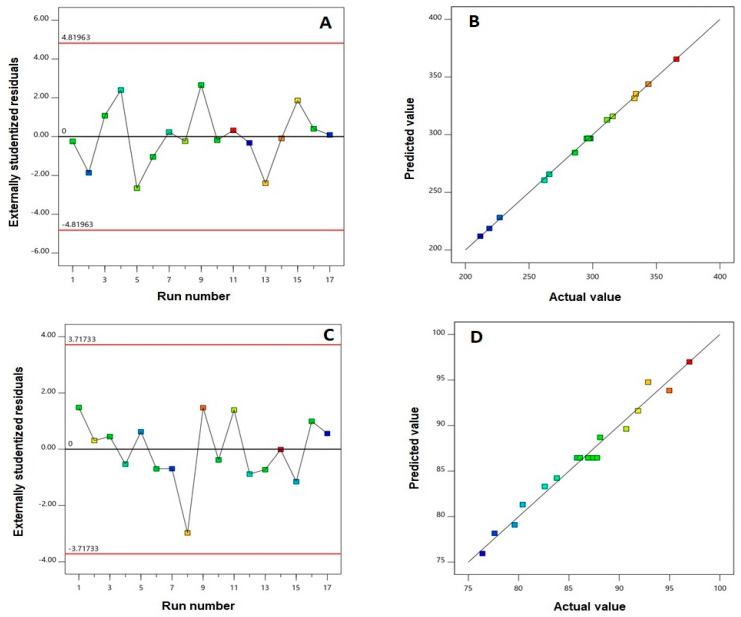
Diagnostic plots for particle size (**A**,**B**) and EE% (**C**,**D**) of AVA PLGA NPs. Externally studentized residuals vs. run number plots (**A**,**C**); and predicted vs. actual value plots (**B**,**D**).

Based on the highest determination coefficient (R2), particle size and EE% of AVA PLGA NPs best fitted the quadratic and linear models, respectively. Statistical output revealed that the predicted R2 value was aligned with the adjusted R2 value for each response, as indicated in Table 3. Adequate precision with a ratio greater than the desirable value of four indicates an adequate noise to signal ratio, and accordingly highlights the suitability of the selected models to explore the design space [25]. Diagnostic plots for the investigated responses are presented in Figure 2, in order to ensure the goodness of fit of the used model and affirm its significance.

### 3.1. Effect of Variables on Particle Size

Particle size of the prepared NPs ranged from 211.7 ± 2.87 to 365.8 ± 4.98, Table 2. The selected model (quadratic) was significant (Model F-value = 1325.08; *p* < 0.0001). There is only a 0.01% chance that an F-value could be this large due to noise. The sequential model equation relating the response to the variables was generated as follows:

Y1 = 296.68 + 57.13 X1 − 19.65 X2 + 5.45 X3 + 4.62 X1X2 + 0.725 X1X3 + 3.93 X2X3 − 8.78 X12 + 5.37 X22 − 7.43 X32(2)

**Figure 3:**
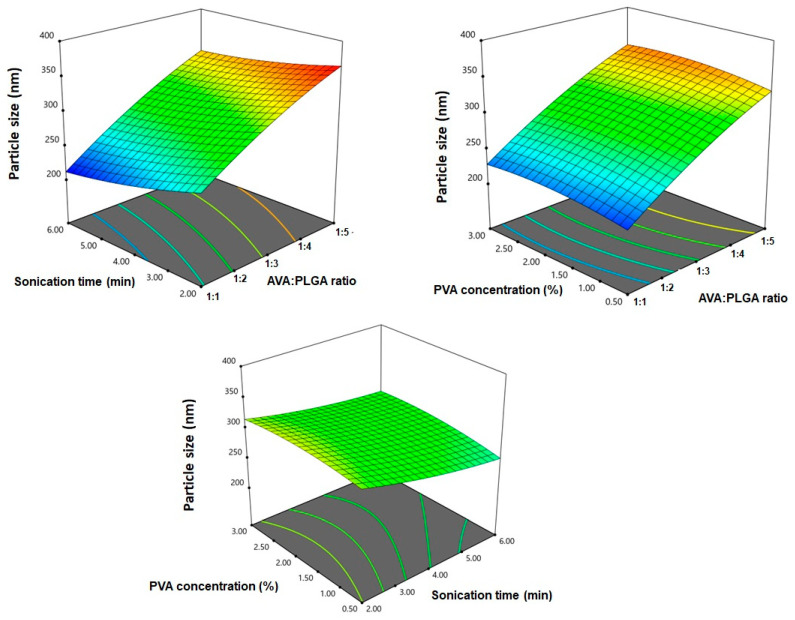
Three-dimensional surface plots for the effect of drug to PLGA ratio (X1), sonication time (X2) and PVA concentration (X3) on the particle size of AVA PLGA NPs.

Statistical analysis of the results using ANOVA revealed that all the investigated factors exhibited a significant effect on the particle size, as evidenced by a *p*-value of < 0.0001 for the linear terms X1, X2 and X3. In addition, the interaction terms X1X2 and X2X3, corresponding to the interaction between the sonication time and either of AVA:PLGA ratio or PVA concentrations, were significant (*p* = 0.0007 and 0.0017, respectively). The quadratic terms X22 and X32, corresponding to the sonication time and PVA concentrations, were also significant (*p* < 0.0001). Figure 3 illustrated three-dimensional surface plots for the effect of the variables on particle size.

### 3.2. Effect of Design Variables on EE%

EE% of the prepared NPs ranged from 77.6 ± 1.08 to 96.9 ± 1.49, as shown in Table 2. The selected model (linear) was significant (Model F-value = 195.53; *p* < 0.0001). There is only a 0.01% chance that an F-value could be this large due to noise. The sequential model equation relating to the response to the variables was generated as follows:

Y2 = 86.46 + 2.69 X1 − 0.4613 X2 + 7.84 X3(3)

**Figure 4:**
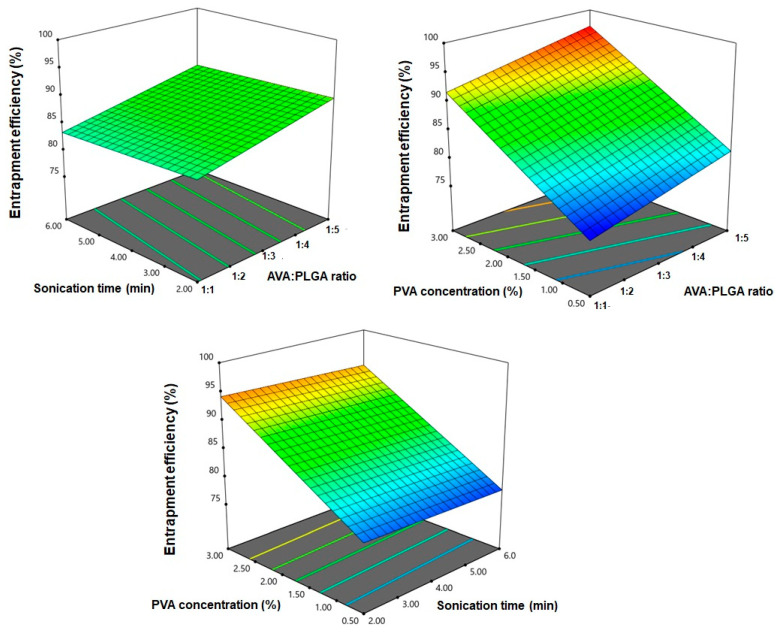
Three-dimensional surface plots for the effect of drug to PLGA ratio (X1), sonication time (X2) and PVA concentration (X3) on EE% of AVA PLGA NPs.

The results of ANOVA statistical analysis revealed that both the AVA:PLGA ratio (X1) and PVA concentration (X3) have a significant effect on EE% (*p* < 0.0001). Figure 4 illustrated three-dimensional surface plots for the effect of the variables on particle size.

### 3.3. Optimization of AVA PLGA NPs

Applying the vesicle size and EE constraints, the optimized levels of the variables were predicted with an overall desirability of 0.843. The optimized formulation was prepared and evaluated. The percentage error between the predicted and observed responses was relatively low, showing that the optimization technique was valid. The variable levels and predicted and observed responses for the optimized formulation are depicted in Table 4.

### 3.4. Optimized AVA-NPs SEM Morphology

**Figure 5:**
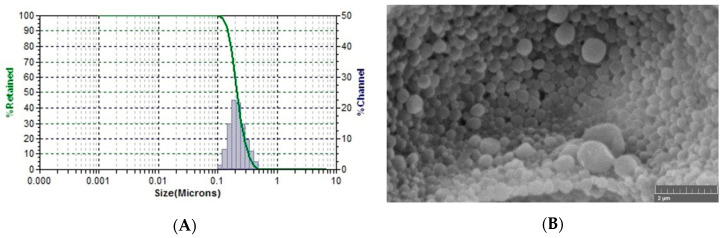
Particle size distribution (**A**) and SEM image (**B**) of the prepared optimized AVA-NPs formulation.

The prepared AVA-NPs revealed particle sizes as measured by particle size analyzer showed a size of 217.4 ± 3.2 nm (Figure 5A). In addition, the SEM image, shown in Figure 5B, revealed that the optimized formulation showed spherical NPs with smooth surfaces, with a relative particle size compared to the data obtained by the particle size analyzer.

### 3.5. In-Vitro Diffusion Study of Optimized AVA-NPs

**Figure 6:**
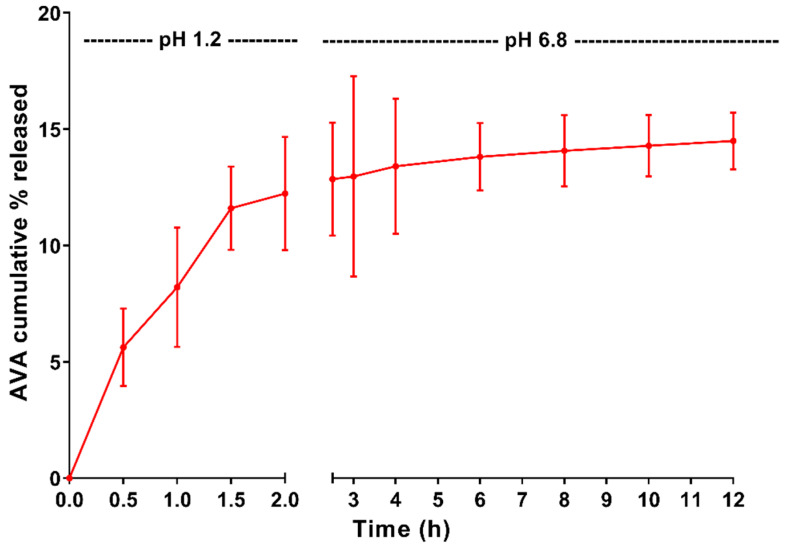
AVA cumulative % released from optimized AVA-NPs formulation.

The release profile of optimized AVA-NPs formulation is shown in Figure 6. The results revealed that AVA-NPs formulation showed a two-phase pH dependent release pattern and an initial fast (burst) phase at pH 1.2 (2 h), followed by a slower pseudo steady state rate pattern phase at pH 6.8 (10 h). The optimized AVA-NPs formulation showed AVA% released of 12.237 ± 2.43% within 2 h of release time at pH 1.2, and 14.498 ± 1.22% within 12 h of release time at pH 6.8. The majority (about 85%) of AVA released from the optimized NPs within 12 h was at pH 1.2.

### 3.6. Clinical Investigation of AVA Formulation in Healthy Human Volunteers

**Figure 7:**
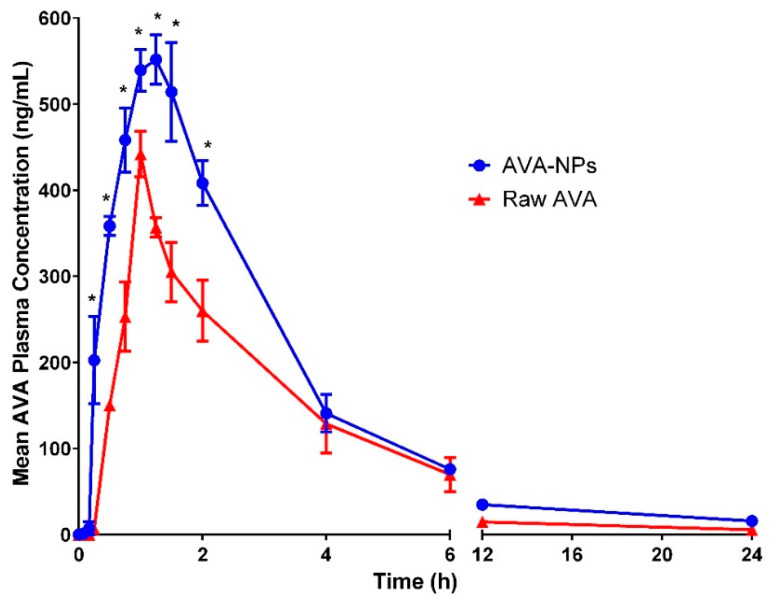
Raw AVA and formulated AVA NPs plasma concentration after single oral dose administration to human volunteers (*n* = 6). * Significant at *p* < 0.05.

The AVA plasma concentration for raw AVA and optimized AVA-loaded PLGA NPs, filled in hard gelatin capsules, after oral delivery in human volunteers (*n* = 6) is shown in Figure 7. In addition, Table 5 illustrates the PK parameters, including C_max_ (ng/mL), T_max_, AUC_0–inf_ (ng h/mL) and T_1/2_ (h), which were analyzed by a PK solver. The results showed that both C_max_ and AUC_0–inf_ were significantly (*p* < 0.05) improved for AVA-loaded PLGA NPs when compared with raw AVA capsules. C_max_ and AUC_0–inf_ for AVA-NPs were 576.3 ± 8.2 ng/mL and 2434.25 ± 179.22 ng/mL*h, respectively (Table 5). The results also revealed no significant change in T_1/2_ and T_max_ for AVA-loaded PLGA NPs relative to raw AVA.

## 4. Discussion

The utilization of nanocarriers in drug delivery have shown promising results for improved drug delivery, targeting, diagnosis and therapy [26,27,28]. This work attempted to enhance the oral bioavailability of AVA through the formulation and optimization of AVA-biodegradable PLGA NPs. The loading of AVA into biodegradable PLGA NPs could enhance the bioavailability of AVA, and also improve AVA efficacy [29]. The design and formulation of AVA-biodegradable NPs requires identifying the formulation and process variables. The application of the Box–Behnken design is of great benefit for its effectiveness in analyzing the influence of various factors. The randomly and uniformly scattered points in the residual vs. run plots illustrated in Figure 2A,C highlight the absence of any lurking variable that could influence any of the responses, and thus confirm the validity of the investigated model [30]. In addition, the normal probability plots of residuals, Figure 2B,D, exhibited a satisfactory linearity confirming the linear distribution of the residuals, and thus the absence for the need of transformation to the data.

The experimental Box–Behnken design results revealed that all the investigated factors exhibited a significant effect on the particle size. The increased PLGA (X1) content leads to an increase in the organic phase viscosity. The size of nanoparticles formed by the evaporation of an organic solvent from the formed emulsion droplets is related to the net shear stress of the sonication, which leads to particle breakdown. The increased viscosity opposes the shear stress, and thus reduces the action of sonication [31]. In addition, the hydrophobic nature of PLGA could interpret the increased size with increased polymer concentration, based on increased polymer association during nanoparticle formation [32,33]. As previously reported, the polymer concentration affects the size of nanoparticles [34,35]. The observed increase in size with an increase in sonication time (X2) could be attributed to the increased temperature of the solution with increased sonication time, which could probably lead to agglomeration of the precipitated PLGA NPs after solvent evaporation. In case of PVA concentration (X3), the observed direct relation of increased size with the increase in PVA concentration could be related to the deposition of PVA on the surface of nanoparticles formed by the organic solvent evaporation from the formed emulsion and nanoprecipitation [36]. Previous reports have indicated the effect of PVA concentration [37,38].

It is important to indicate that NPs showed zeta potential values, measured by the zetasizer, in the range of −3.72 to +1.99 mV. The negative zeta potential value could be related to the presence of AVA molecules (negatively charged) in a deionized aqueous environment. The investigated factors showed no significant effect on the zeta potential prepared PLGA nanoparticles. Accordingly, this is not included in the experimental design investigation. A previous report showed that the zeta potential of PLGA NPs prepared with PVA at pH 7 was 1.87 mV. On the other hand, PLGA NPs at pH 9 showed a high negative value of −24.97 mV, which was attributed to the presence of ionized carboxyl end groups PLGA and ionized hydroxyl groups of PVA on the surface of the prepared NPs [39]. Zeta potential is an important indicator for NPs’ stability, with the increased absolute value of zeta potential indicating improved NP stability with a reduced chance of aggregation [40,41,42]. PVA as a macromolecular emulsifier is used as a stabilizer with a complete surface coating (high surface coverage), due to its high viscosity in the aqueous solution and strong adsorption around the formed emulsion globules [39,43,44]. Accordingly, this improves NPs’ stability and reduces the chance of aggregation by this mechanism.

The AVA:PLGA ratio showed significant (*p* < 0.05) EE% improvement. EE% is affected by polymer concentration, the affinity of drug to organic solvents and the drainage of the drug during solvent removal [12,17,20,21]. The AVA EE% increase with the increase in X1 could be related to the previous finding of increased particles, which indicate direct relation with an increased distance of the AVA diffusional pathways to the external aqueous phase, which reduces AVA loss by diffusion mechanism [35,45]. Additionally, increased PLGA content could increase the viscosity of the organic phase viscosity, which also could hinder AVA diffusion to the aqueous phase, that leads to an increased percentage of AVA entrapped within the polymer matrix of the formed NPs [46,47].

The results of the optimized AVA-PLGA NPs revealed spherical particles with a normal size distribution curve (Figure 5A). This could be attributed to the original content of the emulsion droplets formed during the emulsification process, which affect the size and morphology of the formed (resulted) NPs after solvent evaporation [20,21]. The optimized AVA NPs formulation showed a biphasic release pattern. The initial burst phase is attributed to the release of AVA from the surface (superficial layers) of NPs, as a result of higher concentration of AVA in the outer layers during the drying process. This could also be attributed to the pH of the medium (pH 1.2), which augments the solubilization of AVA and enhanced diffusion from superficial layers of NPs [13]. On the other hand, the second phase, or late pseudo-steady state phase after 4 h, shows a much slower release rate that could be attributed to the AVA release from core (deep) layers of the optimized NP formulation [48]. In addition, the pH of the medium (pH 6.8) could augment slowing the release of AVA from inner NP layers, as a result of reduced solubility compared with an acidic pH environment. After longer periods of time, the hydrolysis of the biodegradable PLGA polymer should contribute to the release of the remaining AVA [49]. According to these results, AVA showed a controlled release pattern from PLGA. In addition, according to previous reports, the release could also be controlled according to PLGA molecular weight used, or the concentration of PLGA in the organic solvent (drug:polymer ratio) [12,20,21,49].

The human pharmacokinetic study of AVA showed a lower initial plasma concentration than that of NPs, possibly due to the slow solubilization rate of raw AVA. After 1.5 h, the plasma concentration for raw AVA was lower than for PLGA NPs, due to the rapid metabolism and elimination of free AVA compared to the encapsulated AVA within the PLGA NPs [50,51]. The findings also revealed that optimized AVA-PLGA NPs significantly changed the pharmacokinetic profile, and improved the bioavailability of AVA by > 1.3 times than that of the raw AVA. This could be related to the fact that AVA is a lipophilic drug with low aqueous solubility, and the formulation of AVA as PLGA-NPs increased not only its solubility, but also the tissue permeability [29,52]. The pharmacokinetic effects of AVA, when administered in the form of PLGA-NP formulation, are also determined by the properties of the primed polymer, rather than by the physicochemical properties of the product molecules.

The improved AVA NPs bioavailability is likely related to the following factors: first, PLGA-NPs introduce AVA as a fine dispersion with a related increased surface area and a reduced diffusion path length, unlike the coarse AVA particles delivered in raw AVA capsules [49,53]; and second, a higher bio-adhesion surface contact between the NPs and the absorption site [54,55]. When the AUC of the prepared AVA NPs was compared with that of raw AVA, the AVA-NPs showed improved values compared to that of raw AVA. This could be attributed to the retention of AVA in the intestinal layers for an extended period, which serves as a drug reservoir for sustained release over several hours. Reports revealed that PLGA moderates the p-glycoprotein effect on absorption and reverses the multidrug resistance activity [55,56]. PLGA augments AVA absorption by its effect on p-glycoprotein though bypassing the p-glycoprotein-mediated efflux induced by a PLGA polymer that enhances oral absorption, as well as the improved bioavailability of AVA [55]. Accordingly, AVA encapsulation within a PLGA NP matrix is a promising method for controlled AVA delivery with improved bioavailability.

In addition, PLGA may have bio-adhesive properties that facilitated AVA absorption through adhesion with the mucosa of the gastrointestinal tract [57]. This results in enhanced AVA bioavailability by increasing residence time [53,54]. The MRT was extended to 9.24 h for AVA-NPs, compared to 5.37 h in the case of raw AVA. In the food industry, PLGA is used as an emulsifier and flavoring agent, as well as an excipient in the pharmaceutical industry, with several advantages when compared to p-glycoprotein inhibitors [56]. These results indicated that AVA PLGA-NPs show a great potential to improve the oral bioavailability of AVA, with more studies to follow. The encapsulation of AVA, a hydrophobic drug, within a PLGA NPs matrix is a promising technique for controlled AVA delivery, with improved bioavailability that enhances its therapeutic efficacy and reduces AVA-related side effects.

## 5. Conclusions

This work utilized AVA characteristics to investigate the optimization and formulation of AVA by emulsion evaporation techniques. This allows the identification of the characters of the produced NPs. The study unfolds the advantage of experimental design to reduce particle size and improve the entrapment of AVA. Controlled release patterns from biodegradable polymeric NPs were also achieved. Furthermore, the significant improvement of AVA bioavailability improves a patient’s compliance, and decreases the dosing frequency of the administered drug.

## Figures and Tables

**Table 1:** Independent variables and responses used in the Box–Behnken design for the formulation and optimization of AVA PLGA NPs.

Independent Variables	Levels		
	(−1)	(0)	(+1)
X1: AVA:PLGA (*w*/*w*)	1:1	1:3	1:5
X2: Sonication time (min)	2	4	6
X3: PVA concentration (%)	0.50	1.75	3.00
Responses	Desirability constraints		
Y1: Particle size (nm)	Minimize		
Y2: EE (%)	Maximize		

Abbreviations: AVA, avanafil; PLGA, poly (lactic-co-glycolic acid); PVA, polyvinyl alcohol.

**Table 2:** Experimental runs and the observed responses of AVA PLGA NPs prepared, according to the Box–Behnken design.

Experimental Run #	Independent Variables			Dependent Variables	
	AVA:PLGA (*w*/*w*)	Sonication Time (min)	PVA Concentration (%)	Particle Size (nm) ± SD	EE (%) ± SD
1	1:3	4.00	1.75	296.4 ± 3.34	87.8 ± 1.76
2	1:1	4.00	3.00	226.9 ± 4.99	91.9 ± 0.98
3	1:3	4.00	1.75	298.3 ± 2.43	86.9 ± 1.11
4	1:1	2.00	1.75	262.1 ± 3.15	83.8 ± 1.21
5	1:3	2.00	0.50	311.3 ± 4.67	79.6 ± 0.86
6	1:3	4.00	1.75	295.3 ± 2.73	85.8 ± 0.91
7	1:3	6.00	0.50	265.9 ± 3.92	77.6 ± 1.08
8	1:3	2.00	3.00	315.7 ± 3.65	92.9 ± 1.89
9	1:3	6.00	3.00	286.2 ± 4.66	94.9 ± 1.88
10	1:3	4.00	1.75	296.5 ± 3.11	86.1 ± 1.45
11	1:5	2.00	1.75	365.8 ± 4.98	90.7 ± 1.99
12	1:1	6.00	1.75	211.7 ± 2.87	82.6 ± 2.14
13	1:5	6.00	1.75	334.1 ± 5.34	88.1 ± 1.77
14	1:5	4.00	3.00	343.8 ± 4.98	96.9 ± 1.49
15	1:5	4.00	0.50	332.8 ± 4.66	80.4 ± 2.11
16	1:3	4.00	1.75	297.4 ± 3.56	87.4 ± 2.31
17	1:1	4.00	0.50	218.8 ± 3.39	76.4 ± 1.29

Abbreviations: AVA, avanafil; PLGA, poly (lactic-co-glycolic acid); PVA, polyvinyl alcohol; #, number.

**Table 3:** Statistical analysis of AVA PLGA NPs responses according to the selected model for each response.

Responses	Model	Sequential *p*-Value	Lack of Fit *p*-Value	R2	Adjusted R2	Predicted R2	Adequate Precision	Significant Terms
Y1: Particle size (nm)	Quadratic	<0.0001	0.1394	0.9994	0.9987	0.9931	125.69	X1, X2, X3, X1X2, X2X3, X12, X22, X32
Y2: EE (%)	Linear	<0.0001	0.3808	0.9783	0.9733	0.9604	44.73	X1, X3

Abbreviations: AVA, avanafil; PLGA, poly (lactic-*co*-glycolic acid); EE, entrapment efficiency.

**Table 4:** Optimized variables levels of optimized AVA PLGA NPs and its predicted and observed responses.

Variables	X1: AVA:PLGA (w:w) Ratio	X2: Sonication Time (min)	X3: PVA Concentration (%)
Optimum values	1:1	6.00	3.00
	Predicted value	Observed value	Error %
Particle size (nm)	213.19	217.42	1.98
EE (%)	91.15	92.67	1.66

Abbreviations: AVA, avanafil; PLGA, poly (lactic-co-glycolic acid); PVA, polyvinyl alcohol; EE, entrapment efficiency.

**Table 5:** Pharmacokinetic parameters of raw AVA and formulated AVA after oral administration of (50 mg) AVA to human volunteers.

Parameter	Raw AVA	AVA-NPs
**k_e_**	0.12 ± 0.03	0.06 ± 0.01
**t_1/2_**	6.05 ± 1.8	12.14 ± 3.81
**T_max_**	1	1.25 ± 0.25
**C_max_**	441.98 ± 26.7	576.3 ± 8.2 *
**AUC _0-inf_obs_**	1448.86 ± 166.2	2434.25 ± 179.22 *
**MRT _0-inf_obs_**	5.37 ± 0.95	9.24 ± 2.35

* Significantly different at *p* < 0.05, unpaired *t* test with Welch’s correction. Abbreviations: k_e_, elimination rate constant; t_1/2_, half-life time; C_max_, Maximum AVA plasma concentration; T_max_, C_max_ corresponding time; AUC, area under the AVA plasma concentration-time curve; and MRT, mean residence time.
